# A cross-sectional study on the prevalence of antibiotic use prior to laboratory tests at two Ghanaian hospitals

**DOI:** 10.1371/journal.pone.0210716

**Published:** 2019-01-15

**Authors:** Gracious Yoofi Donkor, Ebenezer Dontoh, Alex Owusu-Ofori

**Affiliations:** 1 Department of Medical Laboratory Technology, Kwame Nkrumah University of Science and Technology, Kumasi, Ghana; 2 Department of Clinical Microbiology, School of Medical Sciences, Kwame Nkrumah University of Science and Technology, Kumasi, Ghana; Northeastern University, UNITED STATES

## Abstract

There has been a significant rise in global antibiotic use in recent years. Development of resistance has been linked to easy accessibility, lack of regulation of sale, increased tendency to self-medicate and the lack of public knowledge. The increase in antibiotic misuse, including self-medication, has not been well documented in developing countries. Antibiotic use prior to visiting health facilities has been found to be prevalent in developing countries. It has been identified by some studies to increase the likelihood of missed diagnoses and influence the outcome of bacteriological tests. This study is aimed at determining the prevalence of prior antibiotic use through a cross-sectional survey of patients undergoing laboratory tests at two health facilities in Ghana. Face-to-face questionnaires were used to interview 261 individuals chosen by random sampling of patients visiting the bacteriology laboratory of the hospitals within a two-month period. The questionnaire investigated participant demographic characteristics, knowledge about antibiotics and the nature of antibiotic use. Antibiotic property detection bioassay was performed on patient’s urine sample using a disk diffusion method to accurately determine antibiotic use within 72 hours. Culture results were used as an index to evaluate the effect of prior antibiotic use on bacteriological tests. Out of a 261 participants enrolled, 19.9% (95% CI, 14.9–24.9) acknowledged using antibiotics prior to their visit to the laboratory during the study period. On the contrary, 31.4% (95% CI, 25.7–37.5) of participants’ urine samples were positive for antimicrobial activity. Participants within the age ranges of 20–30, 31–40 and 41–50 years had significantly lower odds of urine antimicrobial activity. Participants who had urine antimicrobial activity were more likely to have no growth on their culture plates than participants who had no urine antimicrobial activity [OR 2.39(1.37–4.18), p = 0.002]. The most commonly used antibiotics were the penicillins, fluoroquinolones and metronidazole. Although, majority of the participant (54.8%) had knowledge of antibiotics, most of them had inadequate information on their proper use. The commonest indications for antibiotic use were aches and pains (30.3%), diarrhoea (43.3%) and urinary tract infections (28.0%). Prior antibiotic use was found to increase the likelihood of obtaining a culture negative result and can affect the outcome of bacteriological tests.

## Introduction

Antibiotic use has risen markedly in recent years. A report by the Centre for Disease Dynamics, Economics and Policy (CDDEP) showed that global antibiotic consumption rose by more than 30% from 2000 to 2010 [1]. According to the report, increased access due to growing incomes and the increased use of antibiotics in agriculture are two major factors that have contributed to the rise in antibiotic consumption [1]. High consumption rates are further worsened in developing countries by the inadequate enforcement of laws limiting non-prescription sale of antibacterial agents [2–4]. Unsurprisingly, the highest increase in global antibiotic consumption was observed in developing countries. In India for example, it is estimated that antibiotics administered in hospitals account for 20% of antibiotic use while community use (prescribed or unprescribed) contributes to the rest [5]. Inevitably, rampant use of antibiotics has had grave repercussions in the form of widespread antibiotic resistance. Today, antibiotic resistance accounts for more than 23,000 deaths a year in the United States alone[6].

Prior antibiotic use amongst patients visiting health facilities has risen, especially in developing countries [2,7,8]. A study in Ghana by Lerbech *et al*. (2014) detected antimicrobial agents in the urine of 64% out-patients reporting to two hospitals [9]. Prior antibiotic use has been associated with several serious consequences in some studies. One of such studies found that prior antibiotic exposure was associated with greater length of stay at hospitals following the onset of Gram-negative bacteraemia complicated by severe sepsis [10]. Another study showed that recent antibiotic treatment prior to tests such as blood culture, could reduce culture sensitivity by as much as 62 to 73 percent [11]. Paz *et al*. 2015, also showed, that prior antibiotic medication can significantly reduce synovial fluid culture positivity by 23% [12]. With antibiotics being one of the most frequently prescribed drugs in Ghana [13,14], prior administration before laboratory investigations could potentially reduce the accuracy of cultures and other laboratory tests. Unfortunately, there is a lack of research concerning the prevalence of antibiotic use before such laboratory tests in Ghana.

We therefore investigated the prevalence of patient antibiotic use prior to bacteriological laboratory tests at two hospitals. Furthermore, we investigated public knowledge and use of antibiotics. Finally, we assessed the influence of prior antibiotic use on culture results.

## Materials and methods

### Ethics statement

Ethical clearance was obtained from the Committee on Human Research Publication and Ethics, School of Medical Sciences, Kwame Nkrumah University of Science and Technology. Permission was also obtained from the administrators of both facilities prior to the study. Study participants were also informed of the study and written consent obtained before being included in the study. Consent was obtained from the parents or legal guardians of underage participants before they were enrolled in the study.

### Study design, site and population

This study was a cross-sectional study conducted from March to April 2017 at 2 health facilities in different regions of Ghana; the Bomso Clinic and St. Dominic Hospital. These two facilities represent major health providers outside of government owned hospitals. The Bomso Clinic is 150-bed privately owned health facility in the Ashanti region, that provides routine and specialist services for inhabitants in and around Bomso. St. Dominic’s Hospital is a 450-bed capacity Catholic-funded hospital in the Eastern Region of Ghana. It serves as a referral point for other nearby hospital and also as a facility for training interns and residents.

Patient interviews were conducted at the front desks of the medical laboratory. Eligible patients were first provided oral and written information on the study. Consenting patients were then interviewed with the questionnaire and requested to provide urine samples. Processing of samples and all test procedures were conducted at bacteriology section of the medical laboratory of the various facilities.

### Study population and sample size

The target population of this study was all patients visiting the medical laboratory to undergo bacteriological laboratory tests within the study period. 100 out-patients were randomly sampled at the Bomso Clinic. 161 patients consisting of 62 out-patients and 99 in-patients were also randomly sampled from the St. Dominic’s hospital. Participant ages ranged from 6 to 94 years.

### Data collection methods

#### Questionnaire interview

The survey was conducted using an anonymous face-to-face questionnaire. Patients visiting the hospital’s laboratory were interviewed with the questionnaire. The questionnaire was developed based upon surveys from earlier literature review and tailored to suit the population and the nature of the research. It consisted of three sections; demographic characteristics, knowledge about antibiotics and nature of antibiotic use.

The *demographics* section examined the characteristics of participants including age, sex, level of education and access to health.

The section concerning *knowledge* about antibiotics and their use, ascertained the participants’ awareness about antibiotics and their use. This section determined participants’ awareness of antibiotics, the antibiotics they were familiar with and had used before, and the indications antibiotics could be used to treat. The pattern of antibiotic use was also assessed by identifying factors such as the preferred source of antibiotics and the antibiotics frequently used by participants.

The concluding section investigated information such as frequency of antibiotic use prior to visiting a health facility and antibiotic use prior to visiting the laboratory.

#### Disk diffusion bioassay

Prior antibiotic use was evaluated by determining antimicrobial activity in the patient’s urine sample, using a disk diffusion bioassay. Urine was the preferred specimen since it has higher concentrations of chemical compounds than plasma or serum [15]. The protocol for the disk diffusion bioassay was developed based on previous similar studies [15–17].

- Sample collection for disk diffusion bioassay:

Participants were given a sterile, dry container to provide a urine sample. They were instructed to collect midstream urine while ensuring as minimal contamination as possible. The container was then labelled with his or her hospital code. Samples for in-patients were collected by ward nurses immediately upon admission.

- Protocol for disk diffusion bioassay:
  - Reference organisms, i.e. *Staphylococcus aureus* (ATCC 25923) and *Escherichia coli* (ATCC 25922), were inoculated unto a plate of Mueller-Hinton agar. This was done by collecting 3–5 well isolated colonies of the reference organism with a sterile wire loop, and emulsifying in 4 ml of sterile physiological saline and the turbidity adjusted to 0.5 McFarland. A sterile cotton tipped swab was used to streak the inoculum onto the surface of the Mueller-Hinton agar.
  - Sterile filter paper discs (6mm diameter) were impregnated with 20 μl patient’s urine using a pipette. The discs were dried at 56° C in an incubator. The discs were then placed on the previously inoculated Mueller-Hinton agar plate, with sterile forceps. Commercial antibiotic discs and filter paper soaked in sterile saline were added to each plate as positive and negative controls respectively
  - After 24 hours’ incubation at 35–37°C, the plates were read. The appearance of growth inhibition around the disc was deemed as evidence of urine antimicrobial activity. Duplicate measurements of the zone of inhibition were made with a ruler and the average was recorded. The diameter was recorded as 0mm when bacterial growth extended unto the disc or there was no visible area of inhibition around the disc.

**Fig 1:**
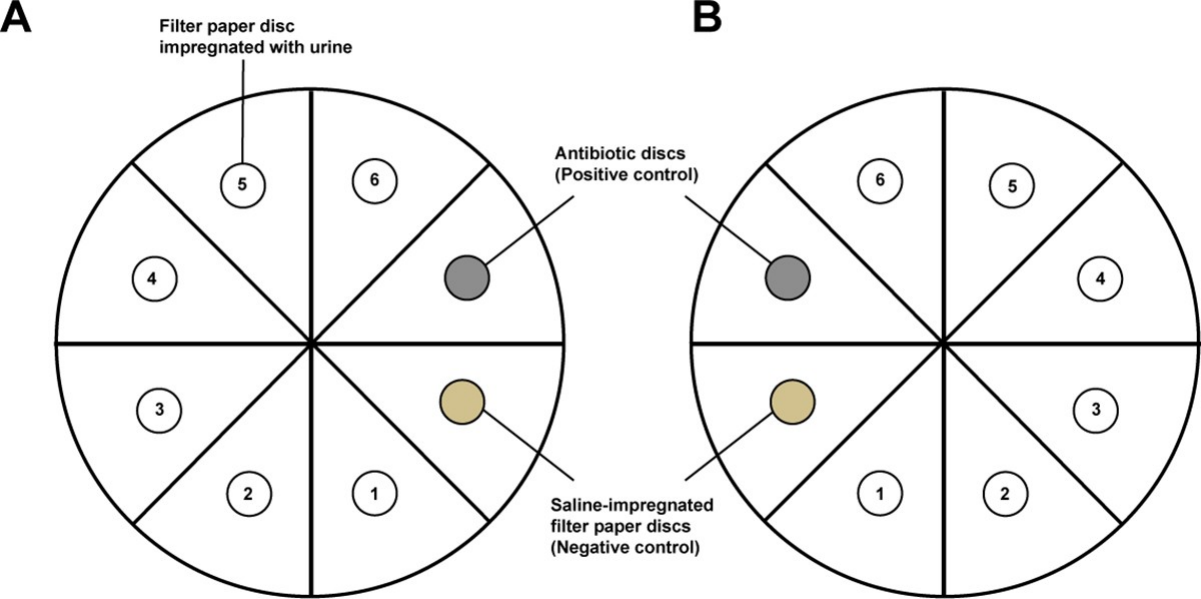
A model for the arrangement of filter paper and antibiotics discs on the agar plates. (A) *Escherichia coli* plate (ATCC 25922) (B) *Staphylococcus aureus* (ATCC 25923) plate.

Each agar plate was used to test for the antibiotic activity in six samples together with the positive and negative controls (Fig 1). This was consistent with the method used by Driscoll et al., 2012, which tested for 2–8 samples on the same plate. As shown in Fig 1, the arrangement and processes were duplicated for each sample on both plates.

### Data analysis

Data collected from the study was entered, coded in Excel and exported into SPSS version22 for analysis. Descriptive statistics such as proportions, means and standard deviation were used to describe the results of the study. The Pearson χ^2^ test was used to assess significant association between the study variables (p<0.05). Logistic regression was used to identify the predictors of urine antimicrobial activity.

## Results

### Socio-demographic profile

Two-thirds of the participants enrolled in the study were female (76.6%). Age distribution was similar across both facilities with majority of the respondents were aged between 20–30 years with a mean age of 36.26 (±SD 14.94). Although, the highest level of education attained by respondents was tertiary education, there were considerably more tertiary educated respondents at Bomso Clinic than at St. Dominic’s Hospital (Table 1).

**Table 1:** Sociodemographic characteristics of study participants.

Category	Health Facility		Total
	Bomso, *N = 100*	St. Dominic's, *N = 161*	*N = 261*
**Sex**			
Female	70 (70)	130 (80.7)	200 (76.6)
**Age**			
<20 years	7 (7)	12 (7.5)	19 (7.3)
20–30 years	38 (38)	52 (32.3)	90 (34.5)
31–40 years	31 (31)	49 (30.4)	80 (30.7)
41–50 years	12 (12)	21 (13.0)	33 (12.6)
51–60 years	6 (6)	11 (6.8)	17 (6.5)
>60 years	6 (6)	16 (9.9)	22 (8.4)
**Department**			
Outpatient	100 (100)	62 (38.5)	162 (62.1)
In-patient	0 (0)	99 (61.5)	99 (37.9)
**Educational status**			
No formal education	3 (3)	12 (7.4)	15 (5.7)
Basic School	11 (11)	27 (16.8)	38 (14.6)
Junior High School	19 (19)	48 (29.8)	67 (25.7)
Senior High School	23 (23)	47 (29.2)	70 (26.8)
Tertiary	44 (44)	27 (16.8)	71 (27.2)
**Relative in Health work**			
Yes	33 (33)	85 (52.8)	118 (45.2)
**Access to health**			
Good	44 (44)	107 (66.5)	151 (57.9)
Moderate	36 (36)	46 (28.6)	82 (31.4)
Poor	20 (20)	8 (4.9)	28 (10.7)

### Knowledge and use of antibiotics

More than half of the respondents (54.8%) had knowledge of antibiotics and the purpose they served (Table 2). About one-third of the respondents were found to have previously used antibiotics while knowing what they were. However, a considerable portion (43.7%) were also discovered to have used antibiotics without knowing what they actually were (Table 2). The most popular source of antibiotics amongst respondents was physician prescription (75.1%) and the most common indications for antibiotics were diarrhoea (43.3%), aches and pains (30.3%) and Urinary Tract Infection (UTI) (23.0%) (Table 2).

**Table 2:** Study participants’ knowledge and use of antibiotics.

Variable	Health Facility		Total
	Bomso, *N = 100*	St. Dominic's, *N = 161*	*N = 261*
**Knowledge of antibiotics**			
Yes	65 (65.0)	108 (67.1)	143 (54.8)
**Used antibiotic**			
Yes	67 (67.0)	26 (16.2)	93 (35.6)
No	3 (3.0)	51 (31.7)	54 (20.7)
Unknowingly	30 (30.0)	84 (52.1)	114 (43.7)
**Source of antibiotic**			
Prescription	78 (78.0)	118 (73.3)	196 (75.1)
Relative advice	6 (6.0)	4 (2.5)	10 (6.2)
Pharmacist	24 (24.0)	0 (0)	24 (14.9)
Self-medication	41 (41.0)	92 (57.1)	133 (70.2)
Advertisement	2 (2.0)	0 (0)	2 (1.2)
On admission	13 (13.0)	0 (0)	13 (8.1)
Left over drug	2 (2.0)	7 (4.3)	9 (5.6)
**Indication for antibiotic**			
Cough	8 (8.0)	30 (18.6)	38 (14.6)
Nasal congestion	26 (26.0)	1 (0.6)	27 (10.3)
Runny nose	19 (19.0)	0 (0)	38 (7.3)
Sore throat	34 (34.0)	1 (0.6)	35 (13.4)
Fever	17 (17.0)	0 (0)	17 (6.5)
Vomiting	4 (4.0)	2 (1.2)	6 (2.3)
Diarrhoea	34 (34.0)	79 (49.1)	113 (43.3)
Aches and pains	35 (35.0)	44 (27.3)	79 (30.3)
Skin wounds	30 (30.0)	41 (25.5)	71 (27.2)
Boils	17 (17.0)	8 (5.0)	25 (9.6)
Urinary Tract Infections	11 (11.0)	62 (38.5)	73 (28.0)
Others	10 (10.0)	6 (3.7)	16 (6.1)

**Fig 2:**
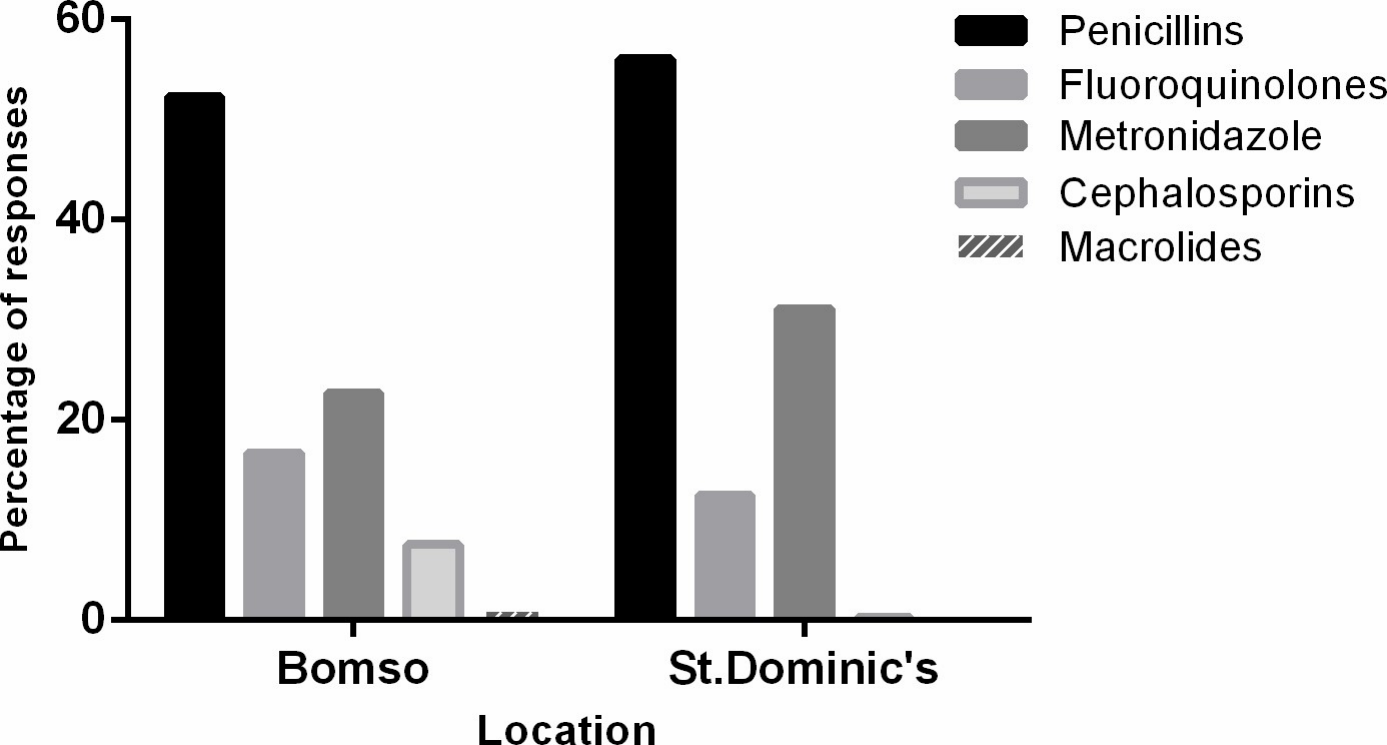
Antibiotic classes used by respondents stratified by location.

Penicillin (i.e. amoxicillin, cloxacillin, flucoxacillin and penicillin) antibiotics were the most commonly used antibiotic in both study locations. The fluoroquinolones and metronidazole were the next most commonly used antibiotics according to the respondents (Fig 2).

### Prior use of antibiotics

One-third of the respondents (33.3%) had used antibiotics before a hospital visit. Out of 261 respondents, 19.9% had used antibiotics prior to their current laboratory visit. About one-third (31.4%) of the respondents tested positive for urine antimicrobial activity. The positivity rate for urine antimicrobial activity was significantly higher at St. Dominic’s hospital than at the Bomso clinic (Table 3).

**Table 3:** Prior antibiotic use stratified by location.

Variable	Health Facility		Total	p-value
	Bomso	St. Dominic's		
**Used antibiotic prior to a hospital visit**				0.177
Yes	28 (28.0)	59 (36.6)	87 (33.3)	
**Used antibiotics prior to** **current visit**				0.1511
Yes	15 (15.0)	37 (23.0)	52 (19.9)	
**Time of prior use**				
1–12 hours	0 (0)	3 (1.9)	3 (1.1)	
12–24 hours	5 (5.0)	29 (18.0)	34 (13.0)	
24–48 hours	3 (3.0)	5 (3.1)	8 (3.1)	
48–72 hours	3 (3.0)	0 (0)	3 (1.1)	
72 hours and above	4 (4.0)	0 (0)	4 (1.5)	
None	85 (0)	124 (47.5)	209 (80.1)	
**Bioassay detected**				**<0.0001**
Positive	14 (14.0)	68 (42.2)	82 (31.4)	

### Culture and sensitivity results

The most commonly isolated organisms were *Escherichia coli* (11.07%) and *Candida spp*. (8.02%), making up 19.09% of the culture results (Table 4).

**Table 4:** Results of participants’ culture tests.

Culture results	Frequency	Percentage(%)
No growth	128	48.85
Insignificant growth	70	26.72
Significant growth	65	24.43
**Isolate**		
*Escherichia coli*	29	11.07
*Staphylococcus* spp	2	0.76
*Proteus mirabilis*	3	1.15
*Klebsiella* spp.	5	1.90
*Enterococcus*	1	0.38
*Enterobacteria*	3	1.15
*Candida spp*.	21	8.02

The majority of antibiotics tested against the isolated organisms (both gram positive and gram negative) were generally ineffective. Majority of isolates were resistant to tetracycline (95.4%), nalidixic acid (90.7%), cefuroxime (85%), cotrimoxazole (93%) and ampicillin (97.7%). The aminoglycosides, amikacin and gentamicin were the most effective antibiotics, showing 25.6% and 54.5% resistance respectively among the total isolates. *Escherichia coli* was most sensitive to amikacin (n = 24), gentamicin (n = 14) and nitrofurantoin (n = 14) and was most resistant to ampicillin (n = 0), cotrimoxazole (n = 1) and tetracycline (n = 1) (Table 5).

**Table 5:** Bacterial isolates and their susceptibility patterns among participants.

		CIP	NIT	AMK	AUG	CXM	GEN	NAL	PPA	CTZ	AMP	TET
Bacterial isolates	N	n (%)	n (%)	n (%)	n (%)	n (%)	n (%)	n (%)	n (%)	n(%)	n (%)	n (%)
E. coli	29	7(24.1)	14(48.3)	24 (82.8)	2(14.2)	5(17.2)	14(48.3)	3(10.3)	7(24.1)	1(3.4)	0(0)	1(3.4)
Klebsiella spp	5	1(20)	1(20)	4(80)	0(0)	0(0)	2(40)	0(0)	1(20)	0(0)	0(0)	0(0)
P. mirabilis	3	2(66.7)	1(33.3)	2(66.7)	1(33.3)	1(33.3)	2(66.7)	1(33.3)	1(33.3)	2(66.7)	0(0)	0(0)
Enterobacteria	3	1(33.3)	1(33.3)	1(33.3)	0(0)	0(0)	0(0)	0(0)	0(0)	0(0)	0(0)	0(0)
Enterococcus spp	1	1(100)	1(100)	0(0)	1(100)	0(0)	0(0)	0(0)	0(0)	0(0)	1(100)	1(100)
Staphylococcus spp	2	1(50)	0(0)	1(50)	1(50)	1(50)	2(100)	0(0)	0(0)	0(0)	0(0)	0(0)

E. coli = Escherichia coli, P. mirabilis = Proteus mirabilis, Klebsiella spp. = Klebsiella pneumonia and Klebsiella spp., Staphylococcus spp = Staphylococcus aureus and Coagulase Negative Staphylococcus, CIP = Ciprofloxacin, NIT = Nitrofurantoin, AMK = Amikacin, AUG = Augmentin, CXM = Cefuroxime, GEN = Gentamicin, NAL = Nalidixic acid, PPA = Pipemidic acid, CTZ = Cotrimoxazole, AMP = Ampicillin, TET = Tetracycline.

### Influence of prior antibiotic use on culture results

Out of the 82 participants who tested positive for urine antimicrobial activity, 58 participants had no growth on their culture plates. Similarly, out of 179 that did not have any antimicrobial activity, 90 were culture negative. Participants who were positive for the bioassay were more likely to have a culture negative result compared to participants who tested negative (OR 2.39 95% CI 1.37–4.18).

### Determinants of antibiotic use prior to laboratory visit

The influence of sociodemographic characteristics of study participants on antibiotic use prior to laboratory visit (as determined by bioassay) was analysed by binary logistic regression at 95% CI. Participants with the age ranges of 20–30, 31–40 and 41–50 years with adjusted odds ratio of 0.29 (0.12–0.97), 0.28 (0.08–0.91) and 0.19 (0.05–0.74) respectively were less likely to have had urine antimicrobial activity. An evaluation of the various levels of education did not show any increased likelihood of prior antibiotic use.

Again, the effect of other factors including knowledge of antibiotics and the source of antibiotics on antibiotic use prior to laboratory visit were also evaluated with binary logistic regression. Although, participants who self-medicated with antibiotics had higher odds of urine antimicrobial activity [1.70(0.88–3.30)], this association was not significant (p>0.05).

## Discussion

Our study found that 19.9% (95% CI, 14.9–24.9) of patients in two Ghanaian health facilities reported using antibiotics prior to their laboratory visit during the study period. This rate was lower than the 31.4% (95% CI, 25.7–37.5) of patients whose urine samples were positive for antimicrobial activity by the bioassay, indicating prior antibiotic use. Comparatively, a similar study conducted by Y. Lui *et al*. 1999, found a slightly lower prevalence of urine antimicrobial activity (25.1%) amongst 203 out-patients in Taiwan [17]. Another study conducted in Northern Uganda showed a much higher prevalence of reported antibiotic use of 62.2% but a similar urine antibacterial activity prevalence of 30.4%. This considerable difference in reported use could likely be as a result of the high prevalence of non-prescription use of antimicrobials in Northern Uganda (75.7%) [18]. Again, another study at two hospitals in Ghana found a higher prevalence of antibiotics in urine (64%)[9]. The significant pervasiveness of prior antibiotic use found in this study and other studies in Ghana could be attributed to the proliferation of antibiotic sources, lax drug regulatory legislation and insidious exposure to antibiotics used in agriculture [19–22]. This increased and inappropriate use of antibiotics has serious implications since it contributes to selective pressure, favouring the development antibiotic resistance [23,24]. Considering that increasing occurrences of antimicrobial resistant bacterial pathogens have been reported in Ghana [25], further studies are required to investigate antibiotic use. This would provide essential information to direct policies on the regulation of antibiotic consumption and the implementation of antibiotic stewardship programmes.

This study found significantly increased culture negative results in patients who were positive for urine antibiotic activity. Participants who had urine antimicrobial activity were more likely to have no growth on their culture plates than participants who had no urine antimicrobial activity. This indicates that prior antibiotic use could increase the likelihood of obtaining culture negative results (no growth on culture plate) and influence the outcome of bacteriological laboratory tests. However, it must be noted that urine antimicrobial activity was not found to have a significant relationship with significant culture results. These findings concur with a study by Paz *et al*. 2015, which found that prior antibiotic administration reduced synovial fluid culture positivity[12] and another study by Arya & Agarwal, which found that the senistivity of culture results was limited in prior antibiotic consumption [16]. This occurrence was attributed to the fact that prior antibiotic use could compromise the recovery of bacterial pathogens and their accurate colony count, resulting in false-negative results and diagnostic dilemmas, especially in symptomatic patients [16].

From the study, it was found that more than half of the study population (54.8%) were aware of antibiotics and the purpose they served. However, most participants reported that antibiotics could be used to treat aches and pains, diarrhoea and UTIs. More of the participants who admitted to using antibiotics for aches and pains were females who reported that they used them for stomach aches or abdominal cramps. These findings point towards an underlying issue of poor knowledge on the appropriate use of antibiotics which could have significant implications on the development of antibiotic resistance.

A little over a third of the study population (35.6%) admitted to knowingly using an antibiotic at some point prior to the study. Disturbingly, 43.7% of the participants had used an antibiotic before but they did so unaware that the drug was an antibiotic. Antibiotic usage was more common at the St. Dominic’s Hospital, where majority of the subjects had low levels of education. This findings highlight the gravity of illiteracy in the increasing inappropriate use of antibiotics in developing countries [26][2]. Penicillin (β-lactam) use far outstripped the use of the other classes of antibiotics amongst participants. According to several studies, penicilins are either the most or amongst the commonly used antibiotics by the public in developing countries [5,18,27–29] and it undoubtedly has serious implication on the emergence of penicillin resistance in such regions. Predictably, resistance to penicillins (β-lactams) is high in Ghana according to studies conducted [30][25].

While a majority of the participants reported that they obtained their antibiotics via physician prescription, a considerable portion admitted to self-medicating with antibiotics at some point prior to the study. Although legislation in Ghana demands that antibiotics be obtained only by prescription, antibiotics are still easily obtained over the counter [29]. This could explain the high rate of self-medication with antibiotics in the study population.

The most commonly isolated organisms were *Escherichia coli* (n = 29) and *Candida spp*.(n = 21). The higher prevalence of *Escherichia coli* is understandable since it is the most common urinary pathogen [31]. More than two-thirds (75.6%) of the cultures performed either showed no growth or had insignificant growth after 48 hours’ incubation. Majority of the isolated organisms were generally resistant against most of the tested antibiotics. The highest resistance was against tetracycline (95.4%), nalidixic acid (90.7%), cefuroxime (85%), cotrimoxazole (93%) and ampicillin (97.7%). The least resistance was found with the aminoglycosides such as amikacin, gentamicin and levofloxacin. This data is consistent with the nationwide surveillance report on antibiotic resistance in Ghana [25].

It must be noted that the study was limited by small sample size and short duration (2 months). Thus the results are not comprehensive enough to make it generalizable. Another limitation was the inability to include in-patients at the Bomso Clinic, which could also have had an effect on the data analysis.

## Conclusion

This study has shown that nearly a third of patients had used antibiotics prior to their bacteriological laboratory tests. It has also shown that prior antibiotic use (as determined by urine antimicrobial activity) increases the likelihood of obtaining culture negative results and thus influences the outcome of laboratory results. These findings confirm the need for health workers to advise patients against using antibiotics before seeking healthcare for any illness. It also re-emphasizes the need for clinicians to obtain their samples for bacteriology cultures before giving antibiotics.

The discrepancy between patients reporting of antibiotic use and the bioassay results shows that patient are unlikely to give an accurate history of their antibiotic use. Thus, a more accurate method such as the disk diffusion bioassay can be used in identifying prior antibiotic use in patients presenting to health facilities. There is the possibility that some patients may not actually know that the drug they had taken was an antibiotic. Furthermore, it may also be possible that patients were afraid of some unknown repercussions if they revealed that they had taken antibiotics. Health care providers should ensure that patients are very relaxed in their presence and should encourage patients to provide accurate information and at the same time reassuring patients that they will not suffer any consequences if they give only the right information.

Penicillins were the most commonly used class of antibiotics amongst patients in the study.

Another important finding of the study was that although a considerable number of the participants were aware of antibiotics and their purpose, they have poor knowledge on the proper use of antibiotics.

## Supporting information

S1 File Study dataset. (XLSX)

S2 File Questionnaire. (DOCX)
