# Supplementary material for: A cross-sectional study on the prevalence of antibiotic use prior to laboratory tests at two Ghanaian hospitals
Source: PLoS One. 2019 Jan 15;14(1):e0210716. doi: 10.1371/journal.pone.0210716 (PMC6333348; doi:10.1371/journal.pone.0210716)
Supplement: S2 File — (DOCX) [file pone.0210716.s002.docx]

**KWAME NKRUMAH UNIVERSITY OF SCIENCE AND TECHNOLOGY**

**FACULTY OF ALLIED HEALTH SCIENCES**

**A CROSS-SECTIONAL STUDY ON THE PREVALENCE OF ANTIBIOTIC USE PRIOR TO LABORATORY TESTS AT TWO GHANAIAN HOSPITALS.**

This questionnaire is aimed at investigating the prevalence of antibiotic use prior to laboratory testing, specifically bacteriology tests. Your submission will form part of the data that will facilitate the realization of this aim.

**DEMOGRAPHICS**

1. Hospital Code:
2. Sex:
3. How old are you? years
4. Describe your educational background. *Tick all that apply

| Level |  |
| --- | --- |
| No formal education |  |
| Basic School |  |
| Junior High School |  |
| Senior High School |  |
| Tertiary |  |
| Other |  |

1. Do you have at least one family member working in a health related field?

Yes No

1. How would you describe your access to health facilities?

| Very Good | Good | Moderate | Poor | Very poor |
| --- | --- | --- | --- | --- |
|  |  |  |  |  |

**KNOWLEDGE ON ANTIBIOTICS AND THEIR USE**

1. a. Do you know antibiotics?

Yes

No

b. If yes, give examples

1. Have you ever taken antibiotics?

Yes

No

1. Which antibiotic have you taken before?
2. How did you obtain the antibiotic?

- Physician prescription
- Self-medication
- Advice from a relative
- Pharmacist advice
- Advertisement
- Hospital
- Left over from previous prescription

1. For which of the following conditions should an antibiotic be used?

- Cough
- Nasal congestion
- Runny nose
- Sore throat
- Fever
- Vomiting
- Diarrhoea
- Aches and pains
- Skin wounds
- Urinary Tract Infections

Others (please specify)

1. Should the instructions that come with the antibiotic be checked before using it?

- Yes
- No

1. How should the dosage be selected?

- Based on physician’s prescription
- Based on the seriousness of your condition
- Instructions on package insert
- Pharmacist advice

1. How many antibiotics should be used at a time?
2. When should one stop taking an antibiotic?

- When he/she feels better
- When the medication is finished
- When an adverse reaction is experienced
- When there is no change in his\her condition

**MEDICATION PRIOR TO LABORATORY TESTING**

1. a. Do you take antibiotics before visiting a health facility?

- Yes
- No

b. How frequently do you do this?

| Rarely | Sometimes | Moderately | Often | Very Often |
| --- | --- | --- | --- | --- |
|  |  |  |  |  |

1. a. Have you used an antibiotic recently before your visit to the hospital?

- Yes
- No

b. How did you obtain it?

- Prescription
- Self-medication

1. a. Did you receive a prescription prior to your visit?

- Yes
- No

b. Have you obtained the prescribed drugs?

- Yes
- No

1. Did you take antibiotics before your visit to the laboratory?

- Yes
- No

1. Estimate the time interval between your antibiotic use and your visit to the hospital?

- 1-12 hours
- 12-24 hours
- 24-48 hours
- 48-72 hours
- 72 hours and above
